# Metacognitive strategy use in GenAI-supported academic reading: a qualitative study of postgraduate students in UK higher education

**DOI:** 10.3389/fpsyg.2026.1787647

**Published:** 2026-03-18

**Authors:** Ying Dai

**Affiliations:** School of Education, University of Birmingham, Birmingham, United Kingdom

**Keywords:** ChatGPT, English academic reading, generative AI (GenAI), metacognitive strategies, qualitative research

## Abstract

In recent years, generative artificial intelligence (GenAI) tools such as ChatGPT have been increasingly integrated into academic reading in higher education. Although GenAI can support processing complex academic texts, its effective use requires learners to employ metacognitive strategies to avoid uncritical reliance. However, how second language (L2) learners use such strategies in GenAI-supported academic reading remains underexplored. Situated in UK higher education, this qualitative study examines how 12 postgraduate L2 students employ metacognitive strategies when using ChatGPT for English academic reading. Data from interviews and retrospective reflections were thematically analyzed, while chat logs were used as supplementary descriptive evidence. The findings identify five categories of metacognitive strategies, namely planning, monitoring, evaluating, information management, and debugging. While many strategies align with prior academic reading research, others are specific to the GenAI context, particularly debugging practices such as correcting GenAI errors and developing personalized prompt templates. Differences were also observed across learners with varying language proficiency, especially in verification and prompt refinement behaviors. This study contributes by providing a qualitative account of metacognitive regulation in GenAI-supported academic reading and extending metacognitive strategy frameworks to GenAI-mediated learning environments.

## Introduction

Academic reading is a core skill in higher education and academic research, but it poses considerable challenges for students. Many Students perceive course reading as a difficult and stressful task, particularly when they are required to handle large amounts of material, make appropriate selections, or deal with unfamiliar academic language. It also demands the adoption of new ways of reading and thinking, which are often difficult to acquire (Bergman, 2024). These challenges make academic reading particularly complex, as it places heavy cognitive and linguistic demands on second language (L2) learners.

To address these challenges, previous research has examined the role of metacognitive strategies in academic reading. Metacognitive strategies refer to those used to supervise and regulate one’s cognitive activities, such as checking the difficulty of a task, testing whether information has been understood, or verifying the accuracy of an outcome (Flavell, 1979). These strategies help students manage the demands of academic texts. For instance, Muhid et al. (2020) demonstrated in a quasi-experimental study with English as a Foreign Language (EFL) students that explicit instruction in metacognitive strategies significantly enhanced reading comprehension. Such findings highlight the importance of planning, monitoring, and evaluating as essential tools for supporting learners’ academic reading. This importance is even more pronounced in L2 contexts. For example, a recent meta-analysis of L2 reading-strategy intervention studies involving learners found that instruction targeting higher-order strategies, such as monitoring comprehension, and asking questions, consistently led to substantial improvements in L2 reading outcomes (Yapp et al., 2021). Prior studies therefore emphasize metacognitive regulation as a key contributor to academic reading success.

With the rapid rise of generative artificial intelligence (GenAI) in the field of L2 education, the context of academic reading is undergoing significant change. On the positive side, GenAI tools can provide quick and personalized feedback, helping learners learn from their mistakes and recognize their strengths and weaknesses (Lu et al., 2024; Rong et al., 2025; Yao et al., 2025b). They can also handle basic language issues such as vocabulary and grammar, allowing students to devote more effort to critical and creative thinking (Yao et al., 2025a). On the other hand, GenAI also poses risks. Students may place excessive trust in GenAI-generated content and accept it without critique, which can hinder the development of deeper critical thinking (Zhou et al., 2024). Therefore, it is necessary to examine metacognitive strategies to understand how GenAI is reshaping academic reading and how students can use it effectively. Regarding metacognitive strategies, they are essential in the GenAI environment because they enable learners to regulate their use of GenAI by verifying, refining, and critically evaluating outputs rather than relying on them uncritically (Lin et al., 2025).

However, existing research still leaves several important gaps. First, most studies continue to focus on metacognitive strategies in traditional L2 academic reading contexts with little systematic exploration of how these processes happen in GenAI-supported reading (Boulware-Gooden et al., 2007; Muhid et al., 2020). Second, the influence of learners’ language proficiency on their use of metacognitive strategies when interacting with GenAI has received little attention. Finally, current research is largely quantitative in orientation, which makes it difficult to capture the complex experiences of students interacting with GenAI (Alvi and Gillies, 2023), and thus more qualitative studies are therefore needed to provide deeper insights into learning processes and individual differences. Building on these gaps, this study aims to explore how Chinese L2 postgraduate students enrolled in taught programs in language education at a UK university approach academic reading in a GenAI-supported context. Specifically, it investigates how this particular group of learners employ metacognitive strategies when using GenAI tools to support their English academic reading. To address these aims, the study is guided by the following research question: What are students’ metacognitive strategies in GenAI-supported English academic reading?

## Literature review

### Metacognitive strategies in academic reading

In the late 1970s, John Flavell put forward the idea of metacognition, describing it as an individual’s knowledge and awareness of their own cognitive processes (Flavell, 1979). This foundational definition highlights individuals’ capacity to reflect on and regulate their own thinking processes. Building on this, Flavell (1979) further identifies three interrelated components of metacognition, including metacognitive knowledge, metacognitive experiences, and metacognitive strategies.

Metacognitive knowledge refers to an individual’s awareness and understanding of the various factors that influence cognitive activities and how these factors interact to shape learning processes and outcomes (Flavell, 1979). Metacognitive experience is defined as the conscious cognitive or affective awareness that learners have when they engage with and process information during a cognitive task, such as completing an assignment (Flavell, 1979). As the key components of metacognition, metacognitive strategies are defined by O’Malley and Chamot (1990) as strategies through which learners plan, monitor, and evaluate their learning process. Specifically, these three sub-dimensions, namely planning, monitoring, and evaluation, explain how learners exercise control over their cognition.

Firstly, planning is conceptualized as the process of strategically selecting appropriate methods and efficiently allocating resources to ensure task completion (Qin et al., 2022). Through planning, learners can set clear goals, organize their approach, and manage time and effort more effectively (Ajayi, 2024). Besides, monitoring is about being continuously aware of how well one understands the material and how effectively they are performing a task. This includes practices like checking progress through self-testing while studying (Schraw, 1998). Through monitoring, L2 learners can examine their reading processes and results, which supports self-regulated learning and enhances reading efficiency (Wang et al., 2023). Additionally, evaluating involves assessing the outcomes and overall efficiency of one’s learning process, which can include reviewing goals or reconsidering conclusions when needed (Schraw, 1998). In other words, learners judge whether their learning strategies and results meet their initial goals and decide if they need to revise their plans or make further improvements. Together, these dimensions work in combination to an integrated metacognitive regulation framework that helps L2 learners better manage their L2 learning cognitive processes and enhance learning effectiveness (Shen and Tao, 2025).

In addition to the three dimensions discussed above, a study by Irwan et al. (2023) brought attention to two additional metacognitive strategy dimensions, namely information management and debugging, which may also play an important role in learners’ academic performance. The study examined the metacognitive awareness of Indonesian English as a Foreign Language (EFL) university students within the context of general academic learning. A total of 420 students from a teacher training institute participated in the study. The findings revealed that debugging strategies, which help learners identify and correct comprehension problems, were reported as the most frequently used, whereas information management strategies, such as summarizing and organizing ideas, were the least frequently used. These results highlight the differential use of these strategies among EFL learners and point to their relevance in the context of language learning.

Academic reading texts are often highly complex, making them especially difficult for L2 learners who must meet the demands of both understanding and critically analyzing what they read (Sun, 2020). As a result, academic reading is not a passive process but rather requires learners to actively employ various strategies to plan, monitor, and evaluate their comprehension, making metacognitive strategies essential for successful understanding (Yüksel and Yüksel, 2011). Boulware-Gooden et al. (2007) investigated whether explicit instruction in metacognitive strategies could improve students’ reading comprehension and vocabulary. The authors found that the intervention group, which was taught strategies such as activating prior knowledge, monitoring, and summarizing, made significantly greater gains in both areas than the control group, which received only traditional reading instruction. Specifically, the control group engaged primarily in copying vocabulary definitions, reading the passages without think-aloud monitoring, and answering a few teacher-posed questions. These findings suggest that metacognitive strategies help learners become more active and effective readers, leading to improved academic reading outcomes. These findings suggest that metacognitive strategies help learners become more active and effective readers, leading to improved academic reading outcomes. Evidence from Muhid et al. (2020) further demonstrates that explicit instruction in metacognitive strategies significantly improved students’ reading comprehension in English, confirming that students who were trained to plan, monitor, and evaluate their reading processes performed better than those who were not.

Although the existing empirical studies have confirmed the importance of metacognitive strategies for L2 reading, students’ metacognitive strategy use within the context of GenAI-supported academic writing is still underexplored. The following sub-section reviews the pertinent literature on this topic.

### Metacognitive strategies in GenAI-supported academic reading

With the rapid advancement of GenAI in L2 reading education, metacognitive strategies are becoming especially important in GenAI-supported academic reading contexts (Lin et al., 2025). GenAI tools such as ChatGPT hold significant potential in supporting students’ use of learning strategies, fostering metacognitive reflection, and enhancing self-monitoring within self-regulated learning contexts (Fahrni et al., 2025). Subsequently, Lin et al. (2025) provides empirical support for Fahrni et al.’s (2025) view by conducting a study involving 15 postgraduate students who used the Kimi Chatbot during academic reading tasks. Through screen recordings and interviews, Lin et al. (2025) identified five key metacognitive strategies in GenAI-supported reading, including planning, monitoring, evaluation, support, and prompting. Building on this, Fu and Hiniker (2025) complement the work of Lin et al. (2025) by examining how these strategies evolve over time and across prompt revisions. Fu and Hiniker (2025) conducted a study in which 19 undergraduate students engaged in academic reading tasks using tools such as ChatGPT and Claude. By analyzing students’ GenAI prompts and written reflections through qualitative methods, Fu and Hiniker (2025) found that while students initially progressed toward higher-order thinking including analyzing and evaluating, they gradually reverted to lower-order prompts, indicating a decline in cognitive engagement over time. Moreover, Fu and Hiniker (2025) also confirm that GenAI can facilitate the use of metacognitive and cognitive strategies but caution that without adequate instructional scaffolding, students may become overly reliant on the technology, limiting deeper engagement.

Besides, Studies also highlight that GenAI- supported metacognitive strategies can have both positive and negative impacts on L2 academic reading. On the positive side, GenAI tools supported L2 learners in applying metacognitive strategies such as planning, monitoring, and evaluation. These tools also enhanced reading efficiency, encouraged critical thinking, and fostered learners’ confidence and autonomy during academic reading tasks (Lin et al., 2025). In addition, GenAI fosters students’ learner autonomy by enabling them to take control of their learning. Borge et al. (2024) conducted an exploratory case study of expert interactions with a chat-based GenAI tool and, through qualitative analysis of prompt revisions, questioning of GenAI responses, and independent inquiry paths, provided evidence that GenAI facilitates self-monitoring and strategic adjustment, ultimately prompting metacognitive regulation and support autonomous inquiry. Taken together, the findings from both Lin et al. (2025) and Borge et al. (2024) suggest that GenAI has potential to support metacognitive engagement, foster learner autonomy, and promote deeper cognitive involvement in L2 learning.

On the negative side, however, GenAI- supported metacognitive strategies may lead to challenges such as cognitive overload and passive reliance. Firstly, Lin et al. (2025) found that students struggled to use GenAI for support and prompting when dealing with abstract theoretical concepts, and the vague or repetitive outputs often left them feeling overloaded and confused. Another challenge is that students may assume GenAI tools are improving their learning, even when they are just passively using them without real thinking. Zhou et al. (2024) surveyed 223 university students to examine the relationship between their perceptions of GenAI tools and cognitive outcomes, finding that only perceived ease of use significantly improved critical thinking and problem-solving, and this effect was mediated by self-regulation.

While existing studies have explored how students use GenAI in academic settings, many studies focus on isolated tasks and thus provide limited insight into how learners apply metacognitive strategies in authentic academic contexts, particularly when using GenAI for academic reading. Therefore, more research is needed to explore how postgraduate L2 learners apply and regulate metacognitive strategies in GenAI-supported academic reading tasks in authentic educational settings.

## Methodology

### Context and participants

This study was conducted at a university in the United Kingdom and involved postgraduate students undertaking taught programs focused on language education and related areas. Within this institutional context, English academic reading formed a central component of students’ coursework and dissertation preparation. As part of their program, students were required to engage in extensive academic reading for both taught modules and independent research projects. These reading tasks typically involved a range of academic text types common in applied linguistics and education, including empirical research articles, theoretical and conceptual papers, methodological texts, and, in some cases, policy or curriculum documents. Students engaged with these materials for purposes such as preparing for weekly seminars, completing coursework essays, developing literature reviews, and building conceptual foundations for their dissertation proposals. In this learning environment, students reported using GenAI tools such as ChatGPT to support their academic reading, particularly for clarifying complex concepts or summarizing lengthy texts, as evidenced by the interview data collected in this study. This practice is consistent with the University’s guidance, which permits the use of GenAI tools for learning support while requiring responsible and ethical use in line with academic integrity policies.

A purposive sampling strategy was employed to recruit individuals most likely to provide meaningful insights into the research question. As Tongco (2007) notes, purposive sampling is particularly effective for gathering in-depth information from knowledgeable individuals, especially when time and resources are limited. In this study, recruitment was carried out through two channels. A brief recruitment announcement was first posted in WeChat groups commonly used by Chinese postgraduate students at the university, and additional potential participants were reached through existing peer networks within the student community. Students who expressed interest contacted the researcher directly. Before confirming participation, each student was informed of the inclusion criteria and invited to consider whether they met the requirements. Only those who were actively engaged in English academic reading as part of their postgraduate studies, who had previous experience using GenAI tools to support such reading tasks, and who felt able to reflect on and articulate their reading behaviors were included. This process ensured that the final sample consisted of participants with relevant and sufficient experience in GenAI-supported academic reading.

A total of 13 participants were initially recruited via the social platform WeChat. One participant withdrew after the initial stage. The final sample comprised 12 participants. All participants were Chinese students whose first language is Mandarin Chinese, enrolled in two taught postgraduate programs in the field of language education including MSc TESOL and MA Education. Pseudonyms (P1–P12) were assigned to ensure anonymity. Although all participants were studying at the postgraduate level in the UK, they differed in several important background characteristics, including age, length of stay in the UK, English level, program of study, and their reported starting point of GenAI use (see Table 1). Ethical approval was obtained through the School of Education’s ethics procedures at the University of Birmingham.

**Table 1:** Participants’ demographic and linguistic profiles.

Pseudonym	Gender	Age	Length of stay in the UK	IELTS score	GenAI use start time
P1	Male	24	12 months	7.5	Postgraduate study
P2	Female	22	12 months	7	Undergraduate study
P3	Female	23	12 months	7	Undergraduate study
P4	Female	25	12 months	7	Postgraduate study
P5	Female	23	11 months	7	Undergraduate study
P6	Female	23	12 months	7	Postgraduate study
P7	Female	23	10 months	6.5	Postgraduate study
P8	Female	26	9.5 months	6.5	Postgraduate study
P9	Female	23	11 months	6.5	Undergraduate study
P10	Female	24	12 months	6.5	Postgraduate study
P11	Female	23	7 months	6.5	Postgraduate study
P12	Female	23	12 months	6.5	Postgraduate study

### Data collection

To capture a comprehensive picture of students’ metacognitive strategy use during GenAI-supported academic reading, this study collected data from three complementary sources: (1) semi-structured interviews, (2) human-GenAI chat logs, and (3) retrospective reflections. Not all participants contributed every type of supplementary data. Of the 12 participants, five (P1, P4, P5, P7, and P11) provided chat logs and four (P1, P4, P5, and P11) submitted retrospective reflections, with partial overlap between these two groups. The remaining participants contributed only interview data. The interviews therefore served as the core dataset, and the chat logs and reflections were used as supplementary sources to enrich and triangulate the analysis. These methods were chosen to provide both self-reported accounts and behavioral evidence of participants’ learning experiences.

(1) Semi-structured interviews

Semi-structured interviews were used to explore participants’ use of metacognitive strategies when interacting with GenAI tools. An interview guide (see Appendix A for the English version and Appendix B for the Chinese version, which was provided to participants) containing around 19 open-ended questions was sent to participants in advance to allow sufficient preparation. Interviews were conducted virtually via WeChat audio calls in Mandarin Chinese, the participants’ first language. With participants’ informed consent, all interviews were audio-recorded, transcribed using the iFLYTEK Hearing App (version 7.0.4755), and securely stored in a password-protected Microsoft OneDrive folder.

(2) Human-GenAI chat logs

Following each interview, participants were invited to voluntarily submit a short segment of their chat history with GenAI tools during a recent academic reading task. Five participants (P1, P4, P5, P7, and P11; see Table 1 for their demographic and linguistic profiles) provided such excerpts, which typically included prompts for definition clarification, summarization, and brief explanations. Participants anonymized all identifying details before submission. These naturally occurring data provided behavioral evidence that could be cross-checked against interview accounts, enhancing the credibility of the findings.

(3) Retrospective reflections

Immediately after submitting their human-GenAI chat logs, participants recorded a brief (around 2-min) retrospective reflection describing their thought processes and strategic decisions during the GenAI-supported reading task. Four participants (P1, P4, P5, and P11) submitted such recordings. As Lam (2008) notes, learner strategies are initially located in working memory and stored as declarative knowledge, which can often be verbalized. These reflections offered insights into cognitive processes not always captured in interviews or chat logs, adding depth to the dataset.

### Data analysis

Data collected from the semi-structured interviews and retrospective reflections were analyzed using thematic analysis following Braun and Clarke’s (2006) six-phase framework, while the GenAI chat excerpts were examined descriptively and used as supplementary evidence. This approach was selected for its flexibility in identifying, analyzing, and interpreting patterns of meaning across qualitative datasets, particularly when exploring metacognitive processes.

In this study, the unit of analysis was defined as a meaning-bearing segment, such as a single sentence, a conversational turn, or a short excerpt from a chat logs, that conveyed a complete idea relevant to the research questions. All coding was conducted manually. Transcripts and chat logs were annotated directly in Word documents using comments and highlighting, and the assigned codes were recorded in a separate coding sheet for organization and later retrieval. A summary of the coding scheme is presented in Table 2. Deductive coding was guided by *a priori* categories derived from the literature, such as planning, monitoring, and evaluating for metacognitive strategies. Inductive coding was applied within these categories to identify new patterns and refine sub-themes. When novel codes emerged, they were compared against the existing framework to determine whether they represented a new sub-theme or an extension of an existing one. During analysis, several observable behaviors, such as summarizing, reorganizing information, simplifying terminology, and translating, emerged across participants’ descriptions. Although these behaviors are commonly viewed as cognitive strategies, they were classified as metacognitive in this study when they served a regulatory function. According to Azevedo (2005), cognitive operations can take on a metacognitive role when learners use them to monitor their comprehension, regulate cognitive effort, or evaluate the adequacy of their understanding. Therefore, in this analysis, text-processing behaviors were coded as metacognitive strategies only when participants explicitly employed them to check whether a passage was understood, manage task difficulty, or evaluate the effectiveness of their reading processes.

**Table 2:** Coding scheme and samples.

Main categories	Themes	Sub-themes	Code	Example
Metacognitive strategies	Planning strategies	Advance planning and preparation before GenAI use	Reviewing and prioritizing documents	“Because there is a limit on the number of documents that can be uploaded, I would first review them all, then select the ones I need most to upload… I might also arrange them in order of priority.” (P1, interview response)
			Preparing materials in advance	“I would definitely prepare the materials I plan to use in advance… and then search for how to give it instructions in a way that is clearer and more accurate.” (P2, interview response)
			Assessing article difficulty before use	“Beforehand, I would assess whether the article is highly theoretical, and prepare for translation, summarizing the evolution of concepts, and explaining complex sentence structures.” (P6, interview response)
			Planning targeted GenAI questions	“I would first consider whether the article reaches a level of difficulty that requires ChatGPT’s assistance, then plan what I want to ask; I might have it introduce the article’s background, skim through the article, and identify the parts where I need help, such as methodology or cited references, and then have it organize those for me.” (P11, interview response)
		Rapid extraction of overall framework and main ideas	Using GenAI to preview content before detailed reading	“Around October last year… I would first have the GenAI read it so that I could get a general idea, and then I would read the original text.” (P1, interview response)
			Summarizing long or complex texts to identify main ideas	“When the text is particularly long, or spans dozens of pages… I use it to help me summarize the main ideas, so that I can understand the content more clearly.” (P2, interview response)
			Seeking clarity on main message when unsure	“When reading a particular article, they were initially unsure about its main message, so they asked ChatGPT to summarize it.” (P7, retrospective reflection recording)
		Task- or difficulty-specific planning	Pasting or uploading text for GenAI explanation, translation, or summarization	“I look up certain technical terms… copy a section of text… and ask it to summarize it for me.” (P3, interview response)
			Selecting GenAI function based on reading needs (summarize, explain, translate, define)	“When the article is too long, I ask it to summarize it; when I need to understand a paragraph, I ask it to explain it; when there are too many unfamiliar words, I ask it to translate them.” (P5, interview response)
	Monitoring strategies	Checking and refining through follow-up questions	Comparing original text structure with GenAI analysis	“I would first compare the headings in the original text with the analysis provided… to see if they generally match.” (P1, interview response)
			Clarifying paragraph meaning and tracing to source	“I would verify by asking what a certain paragraph means… have it point out the specific section… and then go back to the original text to check.” (P4, interview response)
			Testing reliability with keyword searches and multiple rounds	“Based on our own questions… I would search using keywords… and run multiple rounds of instructions to test the answers.” (P2, interview response)
			Breaking down long texts into smaller questions	“I would summarize by chapter or paragraph, list the key points, and for long texts, break questions into smaller parts to avoid overly general answers.” (P7, interview response)
		Using different ways to present and simplify information	Explaining terms and creating visual aids	“I would explain technical terms… summarize paragraphs… and create structural diagrams to aid understanding.” (P3, interview response)
			Analyzing sentence structure and clarifying terms	“For difficult sentences, I would ask it to analyze and explain… point out the grammatical structure… and clarify key terms.” (P4, interview response)
			Translating and simplifying complex vocabulary	“For challenging vocabulary, I would first translate into Chinese, then explain it in simpler English, and break long sentences into shorter ones.” (P3, interview response)
			Using step-by-step explanations in different languages	“When encountering difficult vocabulary, grammar, or phrases… I would first ask for a simple English explanation, and if unclear, then for an explanation in my native language.” (P12, interview response)
		Keeping track of usefulness and thinking critically	Using GenAI selectively for difficult content	“I only use it for sentences I cannot understand after several readings… if the answer gives me an ‘aha’ moment, it’s effective; if it’s off-topic, it’s not.” (P7, interview response)
			Maintaining independent judgment	“I see ChatGPT as a support tool, not a replacement; real understanding and application still require my own judgment.” (P11, interview response)
	Evaluating strategies	Verifying outputs through original and external sources	Checking GenAI content with original text and trusted sources	“When I found that it fabricated content, I would go back to the original text to verify it, then use Google Scholar or consult my teacher.” (P3, interview response)
		Refining prompts to correct inaccuracies	Rewriting and specifying prompts to improve accuracy	“When the understanding was inaccurate, I would reapply the template, write the instructions in more detail and clearly, and use them to correct its response.” (P2, interview response)
	Information management strategies	organizing and summarizing content	Using GenAI to create structured overviews and summaries for better understanding	“I try to get it to generate a relatively clear structural diagram for me, which helps my understanding.” (P3, interview response)
		breaking down content for focused clarification	Requesting multi-level explanations and concrete examples to clarify difficult terms, concepts, or data	“Usually, the first thing is to get a general understanding of the article… the second is to have it explain some of the more difficult technical terms… I hope ChatGPT can give real-life examples so that I can understand what these technical terms in the literature actually mean.” (P8, interview response)
		deciding what to keep or use	Assessing relevance of literature to research topic	“At the early stage of my thesis, I check whether there is relevant literature… whether these articles are related to each other and use it to help me make concise summaries… if they are not related, I might decide not to discuss them together in the literature review.” (P9, interview response)
			Summarizing and integrating key points for inclusion	“Sometimes I incorporate its summaries or suggestions directly into my PDF documents.” (P8, interview response)
	Debugging strategies	Correcting GenAI errors by replacing content and narrowing the task scope	Verifying and correcting misattributed content	“Sometimes ChatGPT… would present the content of article B as if it were from article A. In such cases, I have to check the original article myself to see what it actually says.” (P8, interview response)
			Restarting or rephrasing queries to reset GenAI output	“I have several ways of dealing with this: the first is to start a new conversation; the second is to check it myself… or to ask the question in a different way.” (P8, interview response)
		Developing and refining personalized prompt templates	Designing and applying structured prompt templates	“For example, I might ask it to imitate some sentences based on a template… If it misjudges, I … template in another form, then write my instructions in more detail and clarity, and use that to correct its answer.” (P2, interview response)
			refining prompts for clarity and comprehension	“When ChatGPT gave me an overly broad outline, …add more prompts, give it clearer instructions, and guide it to analyze specific sections or paragraphs, (P1, interview response)

The six phases were implemented as follows. First, the researcher familiarized themselves with the data by repeatedly reading the transcripts, listening to audio recordings, and reviewing chat screenshots while noting initial impressions. Second, initial codes were generated by coding content relevant to the predetermined thematic categories, as well as identifying any novel ideas beyond the initial framework. Third, the codes were mapped onto the *a priori* themes from the literature, and inductive analysis was employed to generate and refine sub-themes within each theme. Fourth, the themes and sub-themes were reviewed to ensure internal coherence and distinctiveness, with adjustments made where overlaps or gaps were identified. Fifth, each theme and sub-theme was clearly defined and named to reflect its conceptual relevance to the research questions. Finally, a coherent, evidence-based narrative was produced, illustrating each theme and sub-theme with excerpts from interviews, reflections, and chat logs to enhance the credibility and clarity of the findings.

To ensure the validity and trustworthiness of the study, a series of strategies were adopted in line with Lincoln and Guba’s (1985) four criteria for qualitative research including credibility, confirmability, transferability, and dependability. First, credibility was strengthened through a transparent and systematic analytic process. The thematic analysis followed Braun and Clarke’s (2006) six-phase framework, combining deductive coding based on established categories with inductive coding to capture emerging patterns. The constant comparative method (Glaser and Strauss, 2017) was applied throughout, with iterative coding and cross-checking across interviews, reflections, and chat logs. This process allowed ongoing refinement and ensured that themes were firmly grounded in the data. Second, confirmability was supported by rigorous data handling and reflexive engagement. All interviews were transcribed using the iFLYTEK Hearing App (version 7.0.4755), and the Mandarin transcripts were translated into English by the researcher. To ensure translation accuracy, the researcher carried out multiple rounds of careful checking between the Mandarin transcripts and the English translations, with close attention given to preserving the original meaning and avoiding distortion. During this process, translation notes were kept to document decisions and resolve uncertainties. These steps helped maintain the accuracy and trustworthiness of the translated data. In addition, analytical decisions, such as the creation of new codes or revisions to the coding framework, were carefully documented to provide a clear audit trail. Reflexivity was also integrated throughout the analytic process. The researcher kept reflective notes to document personal assumptions, interpretive decisions, and potential biases and revisited these notes regularly to monitor their influence on coding. This reflexive practice helped ensure that the analysis remained grounded in the data. Third, transferability was promoted by offering a rich and detailed account of the research context, participant profiles, and the nature of the GenAI-related tasks. This contextual information allows readers to assess the potential applicability of the findings in other settings. Finally, dependability was ensured through detailed record-keeping of the entire analytic process, including annotated transcripts, coding sheets, and notes on theme development. These materials provide transparency and allow others to review the procedures that underpinned the findings. Through these measures, the study sought to produce findings that are credible, contextually grounded, and analytically rigorous.

## Results

### Planning strategies

Within the category of metacognitive strategies, the first theme identified was planning strategies. Analysis of the transcripts showed that participants’ planning in using GenAI for academic reading could be grouped into three subthemes: “advance planning and preparation before GenAI use,” “rapid extraction of overall framework and main ideas,” and “targeted planning for specific tasks or difficulties” (Table 2). The first subtheme, advance planning and preparation before GenAI use, involved participants taking active measures prior to engaging with the tool. This included selecting and prioritizing documents for upload, preparing necessary materials, and learning how to formulate clear instructions. For example, P1 explained, “Because there is a limit on the number of documents that can be uploaded, I would first review them all, then select the ones I need most to upload… I might also arrange them in order of priority.” Similarly, P6 mentioned, “Beforehand, I would assess whether the article is highly theoretical, and prepare for translation, summarizing the evolution of concepts, and explaining complex sentence structures” (interview responses, Table 2).

The second subtheme, rapid extraction of overall framework and main ideas, referred to using GenAI to quickly grasp an article’s structure and main points before detailed reading. For instance, P2 said, “When the text is particularly long, or spans dozens of pages… I use it to help me summarize the main ideas, so that I can understand the content more clearly” (interview responses, Table 2). Similarly, P7 recounted that when reading a particular article, P7 were initially unsure about its main message and asked ChatGPT to summarize it (retrospective reflection recording, Table 2).

The third subtheme, targeted planning for specific tasks or difficulties, reflected the strategy of tailoring GenAI use to address specific challenges, such as understanding complex terminology, translating difficult passages, or summarizing lengthy sections. For example, P11 stated, “I paste a section of text that I cannot understand and ask ChatGPT to explain its meaning. Sometimes I ask it to define certain key terms” (interview responses, Table 2). Overall, students’ planning strategies ranged from advance preparation before using GenAI, to quickly identifying an article’s structure and main ideas, and to formulating targeted approaches for addressing specific reading challenges.

### Monitoring strategies

Within the category of metacognitive strategies, the second theme identified was monitoring strategies. Analysis of the transcripts showed that participants’ monitoring strategies could be grouped into three subthemes: “checking and refining through follow-up questions,” “using different ways to present and simplify information,” and “keeping track of usefulness and thinking critically” (Table 2). The first subtheme, checking and refining through follow-up questions, involved double-checking GenAI’s outputs against the original source, tracing back to specific sections, and asking step-by-step questions to improve accuracy. For example, P1 noted, “I would first compare the headings in the original text with the analysis provided… to see if they generally match.” Similarly, P2 described running multiple rounds of instructions based on specific keywords to test the reliability of responses (interview responses, Table 2).

**Figure 1:**
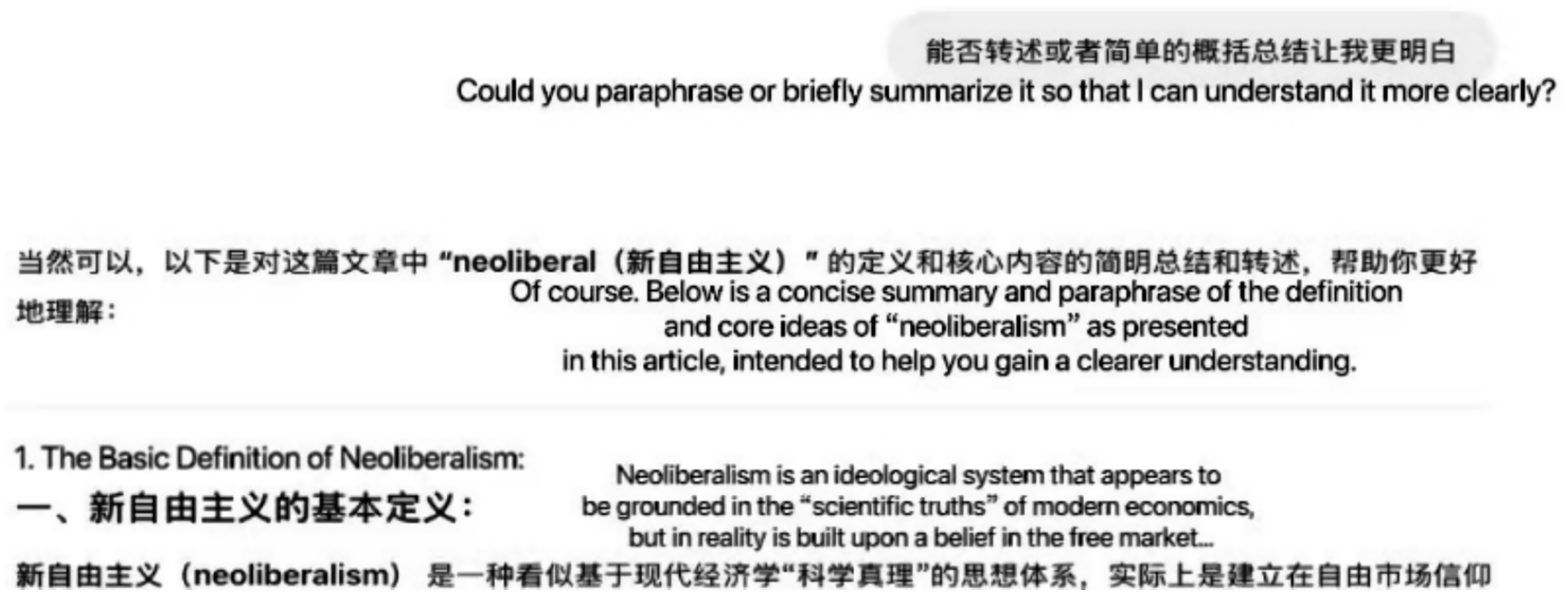
Chatlog screenshot showing participant P4 asking ChatGPT to condense and restate the concept of “neoliberalism” during an academic reading task (translated by the author).

The second subtheme, using different ways to present and simplify information, referred to reformatting or breaking down content to make it easier to understand. Participants described asking GenAI to summarize, explain technical terms, or restructure sentences. For example, P3 explained, “I would explain technical terms… summarize paragraphs… and create structural diagrams to aid understanding.” (interview responses, Table 2). Similarly, P4 asked ChatGPT to restate and condense the definition and core points of “neoliberalism” from a source text into clearer, shorter language. This simplification is shown in the chat log screenshot (Figure 1). Overall, these examples show how students used GenAI to make dense academic content easier to understand.

The third subtheme, judging usefulness and thinking critically, focused on evaluating the quality of GenAI’s answers and maintaining independent judgment. P7 remarked, “I only use it for sentences I cannot understand after several readings… if the answer gives me an ‘aha’ moment, it’s effective. If it’s off-topic, it’s not.” Similarly, P6 emphasized that they did not take answers at face value, and P11 stressed viewing ChatGPT as a supportive tool rather than a replacement for personal understanding (interview responses, Table 2). Together, these three subthemes illustrate how participants verified, adapted, and critically evaluated GenAI’s responses to support their reading.

### Evaluating strategies

A further theme within metacognitive strategies was evaluating strategies, comprising two subthemes: “verifying outputs through original and external sources” and “refining prompts to correct inaccuracies” (Table 2). The first subtheme, verifying outputs through original and external sources, described participants’ tendency to cross-check GenAI’s outputs with original texts and other reliable sources to ensure accuracy. This included returning to the source material, consulting external databases such as Google Scholar or university library systems, and, in some cases, seeking advice from experts. Inaccurate or fabricated information sometimes led participants to abandon GenAI’s output entirely in favor of manual searching or other methods. For example, P3 said, “When I found that it fabricated content, I would go back to the original text to verify it, then use Google Scholar or consult my teacher.” Similarly, P10 recalled, “I once asked it to find literature related to my thesis topic, but some turned out to be fake. When I could not find them in the university library database, I switched to searching directly on the library website” (interview responses, Table 2).

The second subtheme, refining prompts to correct inaccuracies, focused on improving the specificity and clarity of prompts to address GenAI’s errors. Strategies included rewriting or restructuring questions, providing more detailed instructions, adding relevant context, and using alternative phrasing to guide the tool towards more accurate responses. For instance, P2 stated, “When the understanding was inaccurate, I would reapply the template, write the instructions in more detail and clearly, and use them to correct its response.” Likewise, P11 explained, “When explaining novel or niche concepts, if I did not clearly express my needs, it might give a general explanation rather than the field-specific meaning. In such cases, I would ask again with more specificity or provide more context” (interview responses, Table 2). Overall, these evaluating strategies show how participants ensured GenAI’s reliability by verifying its outputs and refining their prompts.

### Information management strategies

Within the category of metacognitive strategies, another theme identified was information management strategies. Analysis of the interview data showed that information management strategies theme could be grouped into three subthemes: “organizing and summarizing content,” “breaking down content for focused clarification,” and “deciding what to keep or use based on processed information” (Table 2). The first subtheme, organizing and summarizing content, referred to processing reading materials in a clear and systematic way, such as outlining, highlighting key points, and producing summaries to quickly grasp the overall structure. For example, P3 explained, “I try to get it to generate a relatively clear structural diagram for me, which helps my understanding.” Similarly, P6 noted that she would ask ChatGPT for the article’s framework, summaries, and term definitions, and then revise them based on their own understanding (interview responses, Table 2).

The second subtheme, breaking down content for focused clarification, described breaking down difficult content into more manageable parts, including defining terms, giving examples, interpreting data, and explaining complex structures to support understanding. For instance, P8 remarked, “Usually, the first thing is to get a general understanding of the article… the second is to have it explain some of the more difficult technical terms… I hope ChatGPT can give real-life examples so that I can understand what these technical terms in the literature mean.” Likewise, P9 commented that they would directly ask ChatGPT about unfamiliar words or complex sentence structures and receive clear explanations (interview responses, Table 2).

The third subtheme, deciding what to keep or use based on processed information, referred to using GenAI-generated summaries, explanations, and analyses to decide whether certain sources should be included in academic work such as a literature review. For instance, P9 explained, “At the early stage of my thesis, I check whether there is relevant literature… whether these articles are related to each other… if they are not related, I might decide not to discuss them together in the literature review” (interview responses, Table 2). Overall, these information management strategies show how students organized and summarized content, deepened understanding through targeted clarifications, and made decisions based on processed information during academic reading.

### Debugging strategies

A further theme identified within the category of metacognitive strategies is debugging strategies. Analysis of the interview data showed that the debugging strategies theme could be grouped into two subthemes: “correcting GenAI errors by replacing content and narrowing the task scope” and “developing and refining personalized prompt templates” (Table 2). The first subtheme, correcting GenAI errors by replacing content and narrowing the task scope, referred to participants’ active measures after detecting GenAI inaccuracies, such as replacing mixed-up or fabricated information using sources personally checked for accuracy, restarting the conversation to reset context, and narrowing the task scope to avoid further errors. For example, P8 described that “Sometimes ChatGPT… would present the content of article B as if it were from article A. In such cases, I must check the original article myself to see what it says.” To correct the errors, p8 noted, “I have several ways of dealing with this: the first is to start a new conversation; the second is to check it myself… or to ask the question in a different way” (interview responses, Table 2).

The second subtheme, developing and refining personalized prompt templates, described participants’ creation and improvement of customized prompt templates to suit their reading habits and comprehension needs. For example, P2 remarked, “I might ask it to imitate some sentences based on a template… If it misjudges, I usually present the template in another form, then write my instructions in more detail and clarity, and use that to correct its answer” (interview responses, Table 2). Participants also demonstrated variation in how they engaged with debugging strategies. Some participants described taking a more active and iterative approach to refining GenAI outputs. For example, P1 explained that “when ChatGPT gave me an overly broad outline, I would add more prompts, give it clearer instructions, and guide it to analyze specific sections or paragraphs,” showing a tendency to restructure prompts and adjust the instructions until the output became more precise. In contrast, other participants reported relying more on GenAI’s initial summaries. As P10 noted, “I usually just let it summarize a long article for me, telling me the main theme and key points,” which reflected a preference for using GenAI as a quick-support tool rather than repeatedly modifying prompts. These differences illustrate the diverse ways in which participants interacted with GenAI (Table 2). Together, these patterns show that debugging strategies encompassed both correcting GenAI errors and developing personalized prompt templates, with participants varying in how actively they refined and reorganized their prompts.

## Discussion

### Planning strategies

The findings revealed that postgraduate students adopted three types of planning strategies when using GenAI for academic reading including advance planning and preparation, rapid extraction of the overall framework and main ideas, and targeted planning for specific difficulties. This represents one dimension of the answer to the first research question on students’ metacognitive strategies in GenAI-supported academic reading. Firstly, students were found to make advance preparations such as screening documents before uploading and designing prompts in advance. This finding is consistent with the general view of planning as strategically selecting appropriate methods and allocating resources (Qin et al., 2022). In this study, planning also involved procedural steps related to interacting with GenAI, such as deciding which files to upload and formulating clear prompts, reflecting the operational requirements of large language model systems, which need explicit and well-structured instructions to generate relevant outputs (Ouyang et al., 2022). In this sense, learners’ planning was reshaped through the tool’s file limits and its reliance on precise instructions, making planning not only about setting goals but also about managing the operational requirements of GenAI tools.

Another strategy was the use of GenAI to quickly identify an article’s structure and key arguments. Obtaining an overview of a text through skimming and previewing can support comprehension (Boulware-Gooden et al., 2007), and GenAI served as an additional tool that facilitated this process. By providing rapid access to the main ideas and organizational structure of lengthy or complex texts, GenAI helped reduce students’ cognitive load. This observation is consistent with recent evidence that GenAI-based tools can scaffold learners’ comprehension of complex academic materials by alleviating cognitive demands during reading (Chen and Leitch, 2024).

In addition, students also planned their GenAI use for challenges, such as unfamiliar terminology, complex sentences, or lengthy sections. This closely reflects Oxford’s (1990) view that learners employ strategies to overcome difficulties in language processing. Rather than relying solely on their own interpretation, students used GenAI selectively when encountering demanding parts of a text, while still engaging in focused reading to make sense of complex ideas. Even with GenAI support, learners still need to focus deliberately on difficult parts. In sum, students’ planning involved goal-setting, previewing, and attending to difficult parts, while also integrating task-specific decisions required when working with GenAI, such as managing prompts, selecting files, and utilizing GenAI-generated support.

### Monitoring strategies

The findings showed that postgraduate students employed three types of monitoring strategies when using GenAI for academic reading including checking and refining through follow-up questions, using different ways to present and simplify information, and judging usefulness and thinking critically. This constitutes another dimension of the answer to the first research question regarding students’ metacognitive strategies in GenAI-supported academic reading. Firstly, students engaged in checking and refining through follow-up questions, such as comparing GenAI’s responses with the original text, tracing back to relevant sections, and using step-by-step prompts to test accuracy. This finding is in line with Lin et al. (2025), who reported that postgraduate students employed GenAI- supported monitoring and prompting strategies to ensure alignment between GenAI responses and their reading purposes. Like our participants, their students re-read uncertain GenAI-generated content, adjusted reading pace, and reformulated prompts to verify comprehension and accuracy. Both studies show that learners use follow-up questions, step-by-step prompting, and source-based comparison to refine GenAI responses and verify their alignment with the original text and reading goals.

Secondly, participants used GenAI to present and simplify information in different ways, including summarizing, rephrasing technical terms, and restructuring dense passages. This finding in a line with evidence from Day et al. (2025), who systematically evaluated how GenAI simplify texts across different grade levels and found that such tools can effectively enhance readability and retain key details. Similarly, postgraduate students in this study relied on GenAI to simplify complex academic readings. A possible reason for this reliance is the high cognitive demand of academic texts. As Sun (2020) notes, academic reading is often dense and conceptually challenging for L2 learners. GenAI tools thus offered a way to reduce this burden. However, Relying on GenAI to simplify complex academic readings also hinted at a potential dependence on GenAI. To address this concern, the next subtheme highlights how students kept independence.

Thirdly, students engaged in critical monitoring by judging the quality and relevance of GenAI’s answers and consciously avoiding over-reliance. This aligns with Zhang’s (2010) view that effective monitoring requires learners to critically evaluate their comprehension and outcomes, rather than passively accepting information. In sum, students in this study felt responsible for judging GenAI’s outputs, showing an active behavior that contrasts with concerns raised by Fu and Hiniker (2025), who found that learners’ engagement may decline over time, leading to more passive reliance on GenAI. Overall, students monitored their reading by checking GenAI’s outputs, simplifying difficult passages, and evaluating their usefulness. These strategies helped them benefit from GenAI’s efficiency while keeping a critical thinking.

### Evaluating strategies

The findings revealed that postgraduate students adopted two main evaluating strategies when using GenAI for academic reading including verifying outputs through original and external sources, and refining prompts to correct inaccuracies. This constitutes the third aspect of the response to the first research question concerning students’ metacognitive strategies in GenAI-supported academic reading. Firstly, the strategy of verifying outputs through original and external sources showed how students cross-checked GenAI’s responses against source texts, library databases, or expert advice. This finding resonates with Schraw’s (1998) definition of evaluation as assessing the results and efficiency of one’s learning process. In this study, evaluation also involved detecting and correcting fabricated information, which sometimes led students to abandon GenAI outputs altogether. This was probably because ChatGPT is prone to generating inaccuracies or even fake references (Yao et al., 2025a).

Secondly, the subtheme of refining prompts to correct inaccuracies illustrates how evaluation was not only about judging the reliability of outputs but also about taking corrective action through improved prompting. This aligns with Borge et al.’s (2024) observation that learners, in their interactions with GenAI, engaged in prompt revisions, questioning of responses, and even independent inquiry paths. A likely reason is the inherent challenge of prompt design, as vague or incomplete instructions can lead to inaccurate outputs, requiring students to refine their prompts (Ouyang et al., 2022). Overall, these evaluating strategies showed that students took active steps, such as detecting fake references, abandoning unreliable outputs, and refining prompts, to ensure that GenAI effectively supported their academic reading.

### Information management strategies

The findings revealed that postgraduate students employed three types of information management strategies when using GenAI for academic reading including organizing and summarizing content, breaking down content for clarification, and deciding what to keep or use. This finding also forms part of the answer to the first research question on students’ metacognitive strategies in GenAI-supported reading. Firstly, organizing and summarizing content in a line with practices reported in Irwan et al. (2023) that learners often relied on their own summarizing and note-taking to structure content. In this study, GenAI was employed to generate initial frameworks and summaries that students subsequently revised based on their own understanding. One possible reason is that GenAI can quickly produce structured outlines and concise summaries, thereby reducing the initial workload of organizing content (Hutapea et al., 2025). This finding extends the concept of information management by showing that in GenAI-supported reading it also involves critically refining GenAI-generated outputs.

Secondly, breaking down complex information into simpler components to clarify meaning expands the scope of information management strategies. Sarıçoban (2015) reported that students often clarified meaning through rereading or by linking new information with prior knowledge. The present study adds to this understanding by showing that students also relied on GenAI to explain technical terms, provide real-life examples, and interpret data. One possible explanation for this is the perceived efficiency of GenAI. Several participants explicitly noted that they found GenAI-generated explanations clearer and faster, which aligns with Kasneci et al.’s (2023) observation that large language models can facilitate comprehension by making complex texts easier to understand.

Thirdly, deciding what to keep or use emerged as a new subtheme. Students used GenAI outputs not only to aid comprehension but also to judge whether certain sources should be included in their literature reviews. This inductive finding extends the scope of information management strategies to academic decision-making. In sum, information management in this study involved using GenAI to organize and summarize content, break down complex ideas for clarification, and make decisions about which sources and ideas to prioritize in academic work. These strategies show that students integrated GenAI as an additional resource to support structuring, clarifying, and selecting information during academic reading.

### Debugging strategies

The findings revealed that postgraduate students employed debugging strategies in two main ways including correcting GenAI errors by replacing content or narrowing the task scope, and developing personalized prompt templates, which also forms part of the answer to the first research question, which examined students’ metacognitive strategies in GenAI-supported reading. Firstly, students engaged in correcting GenAI errors by replacing content or narrowing the task scope. This is similar to Irwan et al.’s (2023) description of debugging strategies, which involve the ability and habit of detecting and correcting errors or adjusting one’s approach. However, in the present study this process also entailed actively checking whether GenAI fabricated references and refining prompts to improve the quality of responses. This difference may be explained by the unique challenges posed by GenAI, such as fabricated references or vague outputs, which require students to intervene strategically rather than simply reprocess information. As Yao et al. (2025a) cautioned, GenAI may fabricate non-existent references, which raises concerns regarding its reliability in academic contexts.

Additionally, an interesting finding is that students used personalized prompt templates as a form of debugging, a strategy not highlighted in prior research on metacognitive regulation. One possible reason is that, as Ouyang et al. (2022) noted, GenAI relies on human feedback to improve its output quality. This reliance may have encouraged students to view prompt refinement as an effective way to optimize their interaction with the system. The data also showed variation in how actively participants engaged in prompt refinement. Some students tended to iteratively adjust and restructure their prompts to obtain more precise or context-specific outputs, whereas others preferred to rely on GenAI’s initial summaries or explanations with minimal modification. These differences reflected individual interaction styles and strategic preferences. Taken together, the debugging strategies observed in this study included both correcting GenAI errors and developing personalized prompt templates, with students differing in how actively they refined prompts during GenAI-supported academic reading.

## Conclusion and limitations

This study aims to explore how postgraduate students employed metacognitive strategies in GenAI-supported academic reading. One research question guided the study: What are students’ metacognitive strategies in GenAI-supported English academic reading?

To address these questions, the study drew on three sources of data including retrospective reflections, human-GenAI chat logs, and semi-structured interviews. Because participants most frequently used ChatGPT, examples focus on their interactions with this tool to illustrate GenAI-supported academic reading. The data were integrated through thematic analysis, which revealed a wide range of dynamic metacognitive strategies. The study identified five main types of metacognitive strategies. Planning included advance preparation before using GenAI, rapid extraction of the framework and main ideas, and targeted planning for specific tasks or difficulties. Monitoring involved refining answers through follow-up questions, simplifying or rephrasing information, and tracking usefulness with critical thinking. Evaluating focused on verifying outputs with original and external sources and refining prompts to correct inaccuracies. Information management covered organizing and summarizing content, breaking down complex points for clarification, and making selective choices based on processed information. Finally, debugging referred to correcting errors by narrowing the task scope and developing personalized prompt templates, with participants varying in how they refined GenAI outputs.

On the one hand, the theoretical contribution of this study lies in addressing key gaps in the literature. It examines how postgraduate students employed metacognitive strategies in GenAI-supported academic reading and identifies how new forms of strategy use, such as personalized prompt templates within debugging strategies, emerged from their interaction with the tool. It also highlights variations in how learners adopted and refined these strategies, demonstrating that students engaged with GenAI-supported reading in different ways. By adopting a qualitative approach, the study further captures learners’ authentic experiences often overlooked in quantitative research, thereby updating learning theories for the age of GenAI. On the other hand, this study provides practical insights for L2 instruction in GenAI-supported academic reading. It shows how teachers can help students adapt and extend metacognitive strategies, such as planning, monitoring, and prompt refinement, while training them in verification to prevent overreliance. The findings also inform higher education practice by suggesting ways to integrate GenAI as structured support that strengthens metacognitive regulation and preserves academic integrity.

Building on these contributions, several pedagogical implications can be drawn for L2 academic reading instruction in GenAI-supported contexts. Teachers can design small, staged reading tasks that guide students through the full cycle of metacognitive strategies, like planning goals, monitoring progress, evaluating reliability, organizing and summarizing information, and debugging errors. Explicit training in prompting skills is also important, such as narrowing the scope of queries, adding contextual details, or rephrasing questions for clarity. To strengthen critical thinking, students may be encouraged to keep simple verification records, for example by quoting original texts or noting differences between GenAI outputs and source materials. In addition, learners should also be guided to maintain independent judgement while drawing on GenAI support. Learners can first attempt independent reading, then use GenAI selectively for clarification or expansion. These practices can ensure that GenAI functions as a supportive tool that enhances metacognitive regulation while preserving autonomy and academic integrity.

This study has some limitations. The participants were all Chinese postgraduate students whose first language is Mandarin Chinese, enrolled in two taught programs in language education at a UK university, so the findings may not reflect wider groups. This study focuses on GenAI-supported academic reading, but since all participants primarily used ChatGPT, the findings are illustrated through this tool, which narrows the tool context. Besides, the data relied on interviews and reflections, which may bring self-report bias, as participants in qualitative interviews may not always respond with full honesty (Shenton, 2004). Moreover, because only five participants submitted chat logs and four provided retrospective reflections, the extent of behavioral triangulation was limited, and the analysis relied primarily on self-reported interview data. Another limitation concerns the uneven availability of supplementary data. While all 12 participants completed the interviews, only five provided chat logs and four provided retrospective reflections, which reduced the extent to which these additional sources could be used to triangulate the interview findings. Finally, while the qualitative approach helped to uncover processes, it cannot exclude other possible factors that may have influenced the findings, such as students’ prior motivation or the impact of their courses and tutors, so the results cannot be attributed solely to GenAI use. Future research could therefore draw on larger and more varied samples, different GenAI tools, and mixed methods to provide a fuller picture.

## Data Availability

The raw data supporting the conclusions of this article will be made available by the authors, without undue reservation.
