# Supplementary material for: Metacognitive strategy use in GenAI-supported academic reading: a qualitative study of postgraduate students in UK higher education
Source: Front Psychol. 2026 Mar 18;17:1787647. doi: 10.3389/fpsyg.2026.1787647 (PMC13038859; doi:10.3389/fpsyg.2026.1787647)
Supplement: Supplementary file 1 [file Data_Sheet_1.docx]

**Appendix A.** **Interview Questions and Retrospective Reflection and Chat Log Submission Guide**

**A: Interview Questions**

To begin with, could you tell me which GenAI tool you use most often when reading for your studies? This could be ChatGPT, or another tool like Gemini or Claude. We’ll base the rest of the interview on your experiences with that tool.

**Section A – General Use of GenAI Tools for Academic Reading**

1. When did you start using GenAI tools to support your academic reading?
2. What kinds of reading tasks do you usually use it for?
3. How do you typically interact with GenAI tools when reading?

**Section B – Metacognitive Strategies**

1. Tell me about your goals when using GenAI tools to support academic reading.

What are you usually hoping to achieve when you turn to GenAI during reading tasks?

1. Before you start reading, how do you usually prepare to use a GenAI tool?

Or can you tell me about any plans you usually make before using GenAI during academic reading?

What kinds of things do you think about in advance, such as when or how to use the tool?

1. Can you describe how you stay aware of how and when to use GenAI effectively while reading?

What helps you decide if you’re using it in a helpful way?

1. Can you describe how you use GenAI to help you understand, organize, or summarize the content of a reading text?

What do you usually do when trying to make sense of what you’re reading with the help of the tool?

1. How do you use GenAI to help you deal with difficult words, phrases, or confusing language when reading academic texts?

Can you give an example of when GenAI helped you better understand the language in a reading?

1. Have there been times when GenAI gave you an unhelpful or inaccurate response?

What did you do then? Can you give an example?

1. What would you say are the main benefits of using GenAI for academic reading?
2. What limitations or frustrations have you encountered when using GenAI for reading?
3. Thinking ahead, if you keep using GenAI for academic reading, what might you do differently to get better results?

Have you learned anything that might change how you use it next time?

**B: Reflection Task (Retrospective Reflection)**

After taking the screenshot of your interaction with the GenAI tool, you will be invited to reflect on your thoughts and experiences during that moment. Please talk about what you were thinking during the task, such as what you understood, what was confusing, how you decided what to ask the GenAI tool, and what strategies you used (for example, planning, checking, or comparing).

There are no right or wrong answers. Just share whatever comes to mind. For example, you might say: “I didn’t understand this part, so I asked the GenAI tool to explain.”

“I was a bit confused by the paragraph, so I asked the tool to summarize it.” “I thought I understood, but I used the tool to double-check.”

You can also talk about how you felt during the task, for example, confident, interested, or a bit lost. This reflection will be audio-recorded by the researcher to capture your thoughts for analysis.

**C: Chat Log Submission Guide**

Chat logs will be collected as a core data source to illustrate the application of metacognitive strategies during GenAI-supported academic reading tasks. Participants will be asked to submit a short excerpt of their GenAI conversation from a reading task. This may include the questions they asked, responses from the tool, and any follow-up prompts. These logs will provide concrete examples of how students plan, monitor, and evaluate their understanding, and how they interact with GenAI in the learning process. The chat logs will be analyzed descriptively. This means they will not undergo thematic coding like the interview and reflection data, but they will be reviewed to identify observable patterns, such as the types of prompts used, the sequence of interactions, and behaviors like clarifying, rephrasing, or asking for more information. The chat logs will serve to triangulate findings from the interviews and reflections, offering concrete, contextualized evidence to support the main themes identified in those datasets. (Participants will be advised to remove or obscure any personal or sensitive information before sharing the logs.)

**Appendix B. Interview Questions and Retrospective Reflection and Chat Log Submission Guide (Chinese)**

A: 访谈问题

首先，能否请您说说在学习中，您最常用的是哪种 GenAI 工具进行阅读？

比如 ChatGPT、Gemini 或 Claude。我们接下来的问题会以您使用该工具的经验为基础进行。

A 部分——GenAI 工具在学术阅读中的总体使用情况

1. 您是什么时候开始使用 GenAI 工具来辅助学术阅读的？

2. 您通常在哪些类型的阅读任务中会使用它？

3. 在阅读时，您通常是如何与 GenAI 工具互动的？

B 部分 —— 元认知策略

4. 请谈谈您在使用 GenAI 工具辅助学术阅读时的目标。

在阅读任务中使用 GenAI 时，您通常希望达成什么样的结果？

5. 在开始阅读之前，您通常会如何准备使用 GenAI 工具？能否分享一下，您在使用 GenAI 进行学术阅读前通常会做哪些计划？比如您会提前考虑哪些事情，例如在什么时候、如何使用该工具？

6. 阅读过程中，您是如何保持对何时以及如何有效使用 GenAI 的意识的？

通常哪些因素会帮助您判断是否在恰当地使用它？

7. 您是如何利用 GenAI 来帮助自己理解、整理或概括阅读材料的内容的？

在借助这个工具来理解文本时，您通常会怎么做？

8. 阅读学术文本时，遇到难懂的词汇、短语或语法时，您是如何使用 GenAI来应对的？

能否举个例子说明 GenAI 是如何帮助您更好地理解语言的？

9. 有没有出现过 GenAI 给出的回答不准确或没有帮助的情况？

当这种情况发生时，您是如何处理的？可以举个例子吗？

10. 您认为在学术阅读中使用 GenAI 的主要优势是什么？

11. 您在使用 GenAI 进行阅读时遇到过哪些局限或让您感到困扰的地方？

12. 展望未来，如果您继续使用 GenAI 进行学术阅读，您可能会在哪些方面调整使用方式，以获得更好的效果？

有没有哪些经验让您考虑在下一次改变自己的使用方式？

B: 反思任务（回顾式反思）1–2分钟

在完成使用生成式人工智能（GenAI）工具支持的阅读任务后，您将被邀请花几分钟时间回顾自己的阅读体验。请谈谈您在完成任务时的想法，比如您理解了什么，哪些地方让您感到困惑，您是如何决定向 GenAI 工具提问的，以及您用了哪些策略（例如：计划、检查、比较等）。

这没有对错之分，只需自然地分享您的真实想法。例如，您可以说：“我不太理解这一部分，所以请 GenAI 工具解释一下。” “我对这段文字有点困惑，所以请工具帮我总结一下。” “我以为自己理解了，但还是用工具确认了一下。”

您也可以谈谈当时的感受，比如是否感到自信、有兴趣，或有些迷失等。

研究人员将对这段反思进行录音，用于后续分析。

C: 聊天记录提交说明

聊天记录将作为本研究的核心数据来源之一，用以呈现学生在 GenAI 支持下进行学术阅读时的行为参与情况及其元认知策略的使用。参与者将被邀请提交一段简短的聊天记录节选，内容包括他们在阅读过程中向 GenAI 工具提出的问题、工具的回复，以及可能的后续对话。这些聊天记录将提供关于参与者如何计划、监控、评估其理解过程的具体例证，并展现他们如何与 GenAI 工具进行互动。聊天记录将进行描述性分析，不同于访谈和反思数据的主题编码处理。研究者将关注可观察到的互动模式，例如使用了哪些提示语、交互的顺序，以及是否出现澄清、重述或进一步提问等行为。这些聊天记录将用于与访谈和反思数据进行三角验证，为研究主题提供具体、情境化的支持证据。参与者在提交聊天记录前，请务必删除或遮盖任何个人或敏感信息。
